# Supplementary material for: Reliability and Validity of the Modified Korean Version of the Chalder Fatigue Scale (mKCFQ11)
Source: Healthcare (Basel). 2020 Oct 24;8(4):427. doi: 10.3390/healthcare8040427 (PMC7712859; doi:10.3390/healthcare8040427)
Supplement: Supplementary file 1 [file healthcare-08-00427-s001.pdf]

Supplementary Table 1. Correlation between the mKCFQ11 biological parameters

Biological parameter (Serum concentration)		mKCFQ11		
		0-week	12-weeks	Changes between 0 and 12-wk
Oxidative stressor	ROS	0.067	0.048	0.235*
	MDA	0.076	- 0.0 28	0.093
Anti-oxidant	TAC	- 0.070	- 0.046	- 0.075
	SOD	0.011	- 0.076	0.031
	Catalase	- 0.117	- 0.133	- 0.143
	Total GSH	- 0.221*	- 0.031	- 0.063
	GSH-Px	- 0.125	0.087	- 0.126
	GSH-Rd	- 0.182	0.062	- 0.059
Cytokine	TNF-α	0.360**	0.099	0.190
	IFN-γ	- 0.240*	0.026	- 0.133

The mKCFQ11: Modified Korean Version of the Chalder Fatigue Scale, ROS: Reactive oxygen species, MDA: Malondialdehyde, TAC: Total antioxidant capacity, SOD: Superoxide dismutase, Total GSH: Total glutathione contents, GSH-Px: Glutathione peroxidase, GSH-Rd: Glutathione reductase. TNF-α: Tumor necrosis factor-α, IFN-γ: Interferon-gamma, The statistical significance of correlation was presented as * $p < 0.005$ and ** $p < 0.001$.
